# Letter to the Editor: Effects of dyclonine mucilage and compound lidocaine cream as tracheal catheter lubricant on postoperative pharyngeal complications after general anesthesia

**DOI:** 10.1097/JS9.0000000000002945

**Published:** 2025-07-07

**Authors:** Minmin Cai, Xiaoya Mao, Xuesi Chen

**Affiliations:** Department of Anesthesiology, the Affiliated People’s Hospital of Ningbo University, Ningbo, Zhejiang, China

*Dear Editor,*

We read with great interest the article by Li *et al* titled “Effects of Dyclonine Mucilage and Compound Lidocaine Cream as Tracheal Catheter Lubricant on Postoperative Pharyngeal Complications After General Anesthesia”^[1]^. This study effectively demonstrates that compound lidocaine cream is more effective than dyclonine mucilage in reducing early postoperative sore throat and improving patient satisfaction. While these are valuable insights, we believe several aspects warrant further discussion. This study is compliant with the TITAN Guidelines 2025—governing declaration and use of AI^[2]^.

Firstly, the original study noted that the benefits of both lubricants diminished or disappeared by 24 hours post-surgery, attributing this to their short duration of action. This presents a significant clinical limitation. Future research should investigate sustained-release formulations, such as incorporating lidocaine into injectable hydrogels or nanoparticles, which have demonstrated the potential for drug release lasting over 4 days^[3]^.

Secondly, the mechanisms by which these agents reduce postoperative cough and hoarseness of voice were largely speculative, primarily attributed to local anesthesia. While local anesthetics like benzonatate inhibit cough by desensitizing pulmonary stretch receptors and blocking nerve impulses, evidence suggests lidocaine may possess anti-inflammatory properties beyond direct neural blockade^[4]^. Intravenous lidocaine has been shown to alleviate acute lung injury by inhibiting the JAK2/STAT3 signaling pathway and downregulating pro-inflammatory cytokines such as HMGB1, IL-6, and TNF-α. In contrast, dyclonine’s primary mechanism is sodium channel blockade, with no clear anti-inflammatory action on tracheal mucosa reported in the available literature. Future studies should explicitly compare the inflammatory marker profiles in tracheal mucosa following dyclonine versus lidocaine application, perhaps through bronchoalveolar lavage fluid analysis or histological examination in animal models^[5]^. This would move beyond speculation and provide a more targeted understanding of their respective benefits.

Thirdly, the intriguing finding regarding foreign body sensation (FBS), where dyclonine mucilage showed a significantly higher incidence of FBS compared to saline, while compound lidocaine cream did not, suggests that the physical properties of lubricants, such as viscosity, texture, and adherence to mucous membranes, may significantly influence patient comfort, potentially independent of or synergistic with anesthetic effects.^[6]^ While a certain viscosity may be beneficial for forming a protective layer, excessive stickiness or a particular texture could itself be perceived as a foreign body. Comprehensive rheological characterization of various lubricants (e.g., shear-thinning properties, adhesion, elasticity) should be undertaken and correlated with detailed patient-reported outcomes and qualitative interviews to understand these nuanced perceptions.

In conclusion, this study provides valuable evidence supporting the use of dyclonine mucilage and compound lidocaine cream as tracheal tube lubricants. By exploring these above avenues, we can refine clinical practices, optimize patient outcomes, and contribute to a more comprehensive understanding of postoperative pharyngeal complications.

## Data Availability

Not applicable.
